# Answer to the letter to the editor concerning “Current state of preoperative embolization for spinal metastasis – A survey by the EANS spine section”

**DOI:** 10.1016/j.bas.2024.104160

**Published:** 2024-12-10

**Authors:** S. Motov, F. Stengel, F. Ringel, O. Bozinov, M.N. Stienen

**Affiliations:** Department of Neurosurgery, Kantonsspital St. Gallen & Medical School of St. Gallen, St.Gallen, Switzerland; Spine Center of Eastern Switzerland, Kantonsspital St. Gallen & Medical School of St. Gallen, St.Gallen, Switzerland; University Hospital Mainz & Johannes Gutenberg-University Mainz, Mainz, Germany; Department of Neurosurgery, Kantonsspital St. Gallen & Medical School of St. Gallen, St.Gallen, Switzerland; Spine Center of Eastern Switzerland, Kantonsspital St. Gallen & Medical School of St. Gallen, St.Gallen, Switzerland

We appreciate the thoughtful comments from Yoshihara et al., which appeared in the 4th edition of Brain & Spine in April this year regarding our survey on preoperative embolization (PE) for spinal metastasis.

Regarding the response rate, we acknowledge this limitation, which we addressed in our original manuscript. However, after thoroughly analyzing the preexisting EANS surveys, we observed a variable average number of responses between 100 and 500. The technical complexity and rarity of PE procedures, as well as the fact that not neurosurgeons but interventional neuroradiologists perform PE in Europe, likely contribute to lower participation rates, a pattern observed in other neurosurgical surveys dealing with specialized topics (Viozzi et al., 2023; Krauss et al., 2024; Hossain et al., 2023).

Moreover, even if 26% of respondents in our survey were residents and only 8.3% stated a higher annual number of spinal stabilization procedures for vertebral metastases, we could not find through multinomial logistic regression analysis any significant influence of the work setting (p = 0.35) and the experience level (p = 0.28) on the use of routine PE. Although the argument for higher-quality data appears legitimate in this context, targeting only board-certified specialists might have led to even fewer responses and reduced the general interest in the survey. Excluding residents from the analysis might have theoretically improved data homogeneity but would have reduced the overall response rates and potentially introduced selection bias by excluding perspectives from training institutions.

The authors also criticized potential institutional bias in the timing of PE. We provided unsuspected data indicating higher availability of PE in public, non-university hospitals than in university institutions. We also commented critically on this finding, mentioning the diverse response rates from different types of institutions (Motov et al., 2023). Regarding the timing of PE, we found that most respondents (64%) requested an embolization either immediately before or within 24 hours before surgery, as recommended in previous literature (Kato et al., 2013; Yuh et al., 2023; Kumar et al., 2016). Whether the PE is performed within this timeframe and what the exact interventionist's workflow looks like was beyond the scope of our survey and is a matter of institutional circumstances that could not be addressed in this publication.

Finally, the authors raised a valid point regarding the risk of spinal cord ischemia due to Adamkiewicz artery embolization. The possibility of PE in specific cases with certain anatomical features has been questioned. There are multiple situations where PE might be too risky, and the advantages are negligible compared to the potentially catastrophic complication of extensive spinal cord infarction. However, we aimed to investigate the current patterns of care regarding PE for surgery of spinal metastases across Europe and not to evaluate every possible scenario for PE. A case-based approach, surveying individual decision-making, might be interesting for future studies. Our results demonstrate that currently, PE appears to be not a routine procedure before surgical treatment of a spinal metastasis.

Without a doubt, there are further specific questions and details about PE that we have not exploited further. This was a conscious decision, as it is well-known that lengthy, time-consuming surveys have a lower participation rate and higher missing data issues (Stienen et al., 2020). Nevertheless, the information that we provided is conclusive and opens the door to further research in this matter.

## IRB approval

Not required for this type of article.

## Declaration of competing interest

The authors declare that they have no known competing financial interests or personal relationships that could have appeared to influence the work reported in this paper.
